# Impact of HPV-16 Lineages Infection in Response to Radio-Chemotherapy in Cervical Cancer

**DOI:** 10.3390/biomedicines11072069

**Published:** 2023-07-23

**Authors:** Fábio Vidal de Figueiredo, Gerusinete Rodrigues Bastos dos Santos, Flávia Castello Branco Vidal, Marcos Antonio Custódio Neto da Silva, Rodrigo Lopes da Silva, Zulmira da Silva Batista, Marcelo Souza de Andrade, Maria do Carmo Lacerda Barbosa, Anna Cyntia Brandão Nascimento Maniçoba, Mayara Cristina Pinto da Silva, Maria do Desterro Soares Brandão Nascimento

**Affiliations:** 1 Post-Graduate Program in Adult Health, Federal University of Maranhão, São Luís 65020-070, Brazil; vidal27@uol.com.br (F.V.d.F.);; 2 Medicine Course, Science Center from Imperatriz, Federal University of Maranhão, Imperatriz 65905-240, Brazil); 3 Medicine I Department, Federal University of Maranhão, São Luís 65020-070, Brazil; 4 University Hospital, Federal University of Maranhão, São Luís 65020-070, Brazil

**Keywords:** cervical cancer, HPV-16 variants, radiotherapy, follow-up

## Abstract

Background: HPV is strongly related to cervical cancer. HPV lineages can contribute to a response to cervical cancer therapy. The aim of this research was to estimate the frequency of human papillomavirus (HPV)-16 lineages in specimens of cervical cancer, relate the pathological factors in these variants, and assess their response to treatment with radical chemoradiotherapy. Methods: Samples of cervical cancer were collected from women who were referred to a reference cancer hospital to test the presence of human papillomavirus-type DNA. The standard protocol of this service consisted of cisplatin-based chemotherapy of 40 mg/m^2^, plus conventional pelvic irradiation in doses of 45–50.4 Gy and high dose-rate brachytherapy of 28–30 Gy to Point A. The response to chemotherapy was evaluated after three months in patients with the HPV-16 lineage. Results: HPV DNA was detected in 104 (88.1%) of the 118 patients. HPV-16 was present in 63 patients (53%). Lineages of HPV-16 were identified in 57 patients and comprised 33 instances of (57.8%) lineage A, 2 instances of lineage B (3.5%), 2 instances of lineage C (3.5%), and 20 instances of (35.0%) lineage D. The median age of the patients was 48.4 years (range 25–85 years). Squamous cell carcinoma was detected 48 times (84.2%). Adenocarcinoma was more likely to occur in lineage D, as three of the four cases occurred in this lineage. A total of 11 patients with the HPV-16 variant were treated with chemoradiotherapy. After three months, it was observed that nine of the eleven patients (81.8%) achieved a complete response, five with the lineage A type, two with the lineage C type, and two with the lineage D type. The two cases of partial response and disease progression, one of each, occurred in lineage A. Conclusions: In addition to the small number of patients and HPV variants, we noticed a better response in patients with the HPV-16 lineage A. Increasing the sample size could be helpful to better assess the impact of HPV variants on cervical cancer treatment.

## 1. Introduction

There were an estimated 8.6 million new cases of cancer and 4.2 million deaths among women in 2018. Cervical cancer occupies the fourth position in incidence and mortality, with 569,847 new cases (6.6%) and 311,365 deaths (7.5%). Its frequency relates to the Human Development Index (HDI), and although its frequency is declining in developed countries, we have observed a burden of new cases in less developed regions. There are expected to be 710,249 new cases in the world as soon as 2030 [1].

Human papillomavirus (HPV) is a non-enveloped virus, measuring 55 nm, whose genome is composed of DNA in a circular double-stranded format. The viral particle, or virion, consists of an outer layer or capsid that surrounds the viral genome. The viral capsid is not surrounded by a lipid membrane, has icosahedral symmetry, and consists of 360 copies (arranged as 72 pentamers) of the major capsid protein L1 and 12 molecules for the minor capsid protein L2 [2,3].

Persistent HPV infection is directly related to the appearance of premalignant lesions and the development of cervical cancer. It is a necessary condition but is not sufficient, as its association with other factors is necessary for the development, maintenance, and progression of intraepithelial lesions [4].

Cervical HPV infection is quite common. It is estimated that 80% of women are infected by the age of 50. About 90% of these infections are naturally eliminated by the body’s immune system within one year [5]. If the infection persists, abnormal cells may appear. Less than 1% of persistent infections lead to invasive cervical cancer [6].

High-risk human papillomavirus (HPV) infection is supposed to be the main cause of cervical carcinoma [7]. Its presence is considered necessary but is not sufficient for the progression of this type of tumor [8]. Among the high-risk HPV types with oncogenic potential, HPV-16 is responsible for half of the cases of cervical cancer [9].

In addition to the classification of HPV types, this infection can also be analyzed in terms of variants. A variant lineage must show a difference between 1% and 10% in the nucleotide sequences of the protein L1. Since 2013, the classification of HPV-16 variants has utilized a numerical alpha pattern and are now grouped into four main strains: A, B, C, and D. Lineage A includes the sublineages that were previously known as European (A1, A2, A3) and Asian (A4). Lineage B comprises the sublineages African 1a and African 1b (B1, B2). Lineage C encompasses the old African 2. Lineage D includes the North American (D1) and Asian American (D2, D3) sublineages [10,11]. Several studies have suggested that this genetic variability between the HPV-16 variants could influence the potential for infection, viral persistence, and the development of invasive cervical cancer [12,13,14]. 

The process that leads a cell infected by HPV to transform into a malignant lesion is a slow process, which is associated with the oncogenic type, the viral load, the affected site, and the persistence of the infection. Low-grade intraepithelial lesions are usually observed in productive viral infections. The progression of these lesions to malignancy is often associated with the active transcription of the viral genes E6 and E7, which promote cell growth and contribute to the immortalization of these cells, with the integration of the viral DNA through the host cell DNA [15,16].

Regarding treatment options, hysterectomy approaches include simple hysterectomy (type A), modified radical hysterectomy (type B), and radical hysterectomy (type C)—the latter being recommended for the treatment of cervical cancer [17]. It is a procedure typically used in early tumors such as IA, IB1, and IIA1 staging [9] (American College of Gynecologists, 2002). Radiotherapy, associated with chemotherapy, is usually the treatment of choice for IB2 up to IVA staging [18,19]. Chemoradiation can also be used in patients who are not candidates for hysterectomy. Radiotherapy plus chemotherapy using cisplatin has demonstrated a 30–50% reduction in the risk of death and an improvement in overall survival and disease-free survival when compared to radiotherapy alone [20].

The role of genotyping HPV to determine a better outcome in cervical cancer treatment remains controversial. Some studies have proposed that the determination of the HPV type has no prognostic consequence in cervical cancer [21], and others have demonstrated a worse response to radiation registered in patients infected with HPV-16 [22]. It was hypothesized that this phylogenetic single-nucleotide polymorphism among HPV-16 lineages and sublineages could provide a difference in the response to cancer treatment with radiotherapy and chemotherapy, depending on the detected HPV variant [23], and thus serve as a prognostic factor in the treatment of cervical tumor. Nevertheless, the function of HPV variants as a modulating factor in their response to treatment in the carcinoma of the cervix is still uncertain.

Due to the importance of this topic on public and women’s health, the present study aimed to determine the prevalence of HPV and investigate the relationship between HPV-16 strains and their response to treatment with chemotherapy, radiotherapy, and brachytherapy in patients with cervical cancer.

## 2. Materials and Methods

### 2.1. Sample

Patients with proven cervical carcinoma were requested to join this study, which was conducted between April 2016 and April 2018 at the Aldenora Bello Cancer Hospital and Tarquinio Lopes Filho Cancer Hospital, located in São Luis, Maranhão (Brazil). 

Samples of tumor material were collected from 125 women with cervical cancer. A follow-up was carried out, and the oncological treatment was recorded, according to the protocols of the hospitals, when the patients returned for a consultation to evaluate the treatment performed.

This study was approved by the Institutional Ethical Committee under the number 1.289.419/2015. All patients were informed and given signed permission to participate before the trial’s initiation.

### 2.2. Epidemiological Data

Patients referred for outpatient care at the gynecological oncologist services at these hospitals were invited to participate in this study. If they accepted the objectives of the study, they signed an informed consent form (ICF) and were given a questionnaire for the collection of socio-demographic data.

The patients in whom the presence of a tumor was demonstrated in a biopsy of the uterine cervix and who met the inclusion criteria were followed up after diagnosis. Treatment data were collected via a clinical questionnaire, which recorded the performed oncological procedures (surgery, radiotherapy, chemotherapy, or other) until their first follow-up return with tests to assess their response to the treatment performed. 

### 2.3. Inclusion and Exclusion Criteria

The inclusion criteria were women over 18 years of age diagnosed with cervical cancer who agreed to participate in this research by signing an informed consent form.

The exclusion criteria were women with small lesions in which the performance of a biopsy could interfere with staging, women undergoing psychiatric treatment, and women who refused to sign the informed consent form. 

### 2.4. Experimental Procedures

The collected samples containing tumor fragments were placed in microtubes containing 1 ml of RNAlater and were transported in thermal boxes at 4 °C and sent to the Multiuser Laboratory located in the Biobank of Tumors and DNA of Maranhão following registration and storage protocols. After 24 h, the sample of RNA was removed and stored in a freezer at −80 °C until the procedures were performed in the laboratory.

The extraction of genomic DNA from frozen samples was performed as per the protocol described by the DNeasy Blood and Tissue Kit user manual (Qiagen, Hilden, Germany).

For the identification of HPV DNA in samples of cervical tumors, the PCR nested amplification technique was used, according to Vidal et al. (2016) [24]. 

The determination of HPV genotypes was performed via the automated sequencing of the PCR product using the MegaBACE 1000 sequencer (GE Healthcare, Chicago, IL, USA). Reactions were carried out at the Molecular Biology Laboratory of the State University of Maranhão, located at the Center for Higher Studies in Caxias (CESC—UEMA). The sequencing was performed using the BigDye™ Terminator v3.1 Cycle Sequencing Kit (Thermo Fisher Scientific, Waltham, MA, USA), according to the manufacturer’s protocol. 

For the analysis and alignment of the nucleotide sequences obtained in the sequencing, the Chromas program was used, which obtained the electropherograms of the HPV DNA sequences present in the samples. For the confirmation and identification of the type of HPV, a comparison of the nucleotide sequences of these samples was performed, and the samples were then sequenced and then submitted to the world nucleotide database GenBank using the BLAST program (NCBI).

### 2.5. HPV Variants

After identifying the types of HPV present, the samples infected by HPV-16 were submitted to a new PCR amplification protocol of regions to the viral genome, which was capable of identifying the variant to which that type of HPV detected belonged.

Two pairs of primers were used, which amplified the entire LCR region and the E6 gene.

The PCR product was further purified and sequenced according to the aforementioned protocol. Consensus sequences were joined using the software Geneious version 2020.1 (Biomatters Ltd.), and all generated sequences were aligned according to the specific strains of HPV-16, using reference sequences proposed by Burk et al. (2013) [11] and using MEGA software (version 6.0, www.megasoftware.net, accessed on 20 September 2018). An analysis of the variants of HPV-16 and the construction of the phylogenetic tree was carried out at the National Institute of Cancer under the supervision of Dr. Miguel Angelo Martins Moreira.

### 2.6. Clinical Data

The staging system of the International Federation of Gynecology and Oncology (FIGO) was used to classify all patients. Contrast-enhanced computed tomography (CT) of the abdomen and pelvis was performed to assess lymph node involvement. Pathological features, such as tumor size, histological type, the grade of differentiation, hemoglobin levels, and parametrial and vaginal features, were recorded. We included patients treated with concurrent chemotherapy radiotherapy treatment (CCRT) for the purpose of identifying a cure.

Patients were treated according to the department protocol. The external beam radiotherapy (EBRT) was performed with a 4-field box procedure to the whole pelvis, protecting organs at risk, with prescript 45–50.4 Gy doses, daily fractions of 1.8 Gy, and five weekly fractions. An additional parametrial boost up to 54 Gy, protecting the midline, was provided with a dose planned according to the stage. External beam irradiation was executed using a Cobalt-60 or 6 MV linear accelerator (Siemens). The patients under CCRT treatment received cisplatin, 40 mg/m^2^ for 6 cycles weekly, with concurrent external radiation. Intracavitary brachytherapy was provided, employing a high-dose-rate (HDR) after the loading technique, which was supplied in four fractions, two times a week, with doses of 7–7.5 Gy per session to Point “A”, according to the clinical stage and tumor volume.

### 2.7. Local Response to Treatment

The assessment of the local response to treatment was programmed to occur 12 weeks after the end of radiation, performed through a gynecological clinical examination and cervical cytology, as well as CT in all cases (with a necessary ultrasound or magnetic resonance imaging whenever needed) and cervical biopsies in uncertain cases, in order to assess the absence or presence of a tumor. They were classified as a complete response (a 100% decrease in the original tumor volume without the clinical or radiologic presence of disease), partial response (a decrease in the original tumor volume by at least 50%), stable disease (a reduction of less than 25% or progression of up to 10% of the initial volume), and the progression of disease (an increase of more than 25% of the initial volume).

### 2.8. Ethical Aspects

This study complied with all the principles set out in the Declaration of Helsinki and Resolution 466 from December 2012 of the National Health Council of the Ministry of Health, guaranteeing the secrecy, reliability, and dignity of the subjects in the research, as well as guaranteeing their autonomy and the defense of their vulnerability.

The study project was approved by the Research Ethics Committee of the Federal University of Maranhão (CEP-UFMA) under Consolidated Opinion No. 1.289.419/2015.

### 2.9. Statistical Analysis

Descriptive statistical analysis was performed using the Stata program (version 14.0), with data presented in the form of figures and tables. To evaluate the association between the HPV and sociodemographic and clinical variables, the χ^2^ (squared) test was used, and *p*-values of ≤ 0.05 were considered statistically significant. 

The values referring to “does not know/did not answer” were excluded from the association analysis.

## 3. Results

### 3.1. Sociodemographic Data

The study population consisted of 118 women who were diagnosed with cervical cancer and treated at the Aldenora Bello Cancer Hospital and Tarquínio Cancer Hospital Lopes Filho. 

The age range varied between 25 and 89 years, with an average age at diagnosis of 50.6 years old. Most of the cases, around 75 (63.5%), occurred between 30 and 59 years of age. Most women declared themselves to be Brown (69.5%). Women who were married or in a stable union predominated (51.7%) 

Smoking was a habit reported by about 50 women (42.4%). The largest contingent of women were illiterate (33.0%), and only 22 women (18.6%) had 12 or more years of education. The per capita income in the families of 76 women (64.4%) was less than half the minimum wage (corresponding to an amount of BRL 880.00 at the time of the study). Most of the women, 52 (44.1%), came from municipalities with a low human development index (HDI).

### 3.2. Reproductive Data

When evaluating the patients’ reproductive histories, it was observed that 75 women (63.6%) started sexual activity before the age of 18, and 63 women (53.4%) reported having up to two sexual partners throughout their lifetime. High parity was observed, with 62 women (52.6%) having more than five children. Most women, 87 (73.7%), reported having knowledge of birth control; 15 women (12.7%) reported not using birth control, while 48 women (40.7%) had used it regularly or irregularly over the last 3 years (Table 1).

### 3.3. Clinical Data

There was a high frequency of advanced stages at diagnosis. EC IIB was recorded in 30 women (25.4%) and EC IIIB in 50 women (42.4%). Squamous cell carcinoma was the most frequent, with 94 cases (79.7%), while adenocarcinoma was observed in 11 cases (9.3%). About 67 patients (56.8%) had a tumor with a diameter greater than 4.0 cm. The presence of lymph node metastasis was observed in 12.7% of cases (Table 2).

Regarding the treatment performed, it was observed that surgery (either followed or not by an adjuvant with radiotherapy and/or chemotherapy) was the procedure performed on 25 women (21.1%), most of whom were in their initial clinical stage. Treatment with radiotherapy and chemotherapy, alone or in combination, was used for 64 women (54.2%), most of whom were in advanced clinical stages. A total of six patients (5.0%) underwent treatment in another city, and about twenty women (16.9%) did not undergo treatment.

### 3.4. HPV Types and Lineages

The HPV test was positive in 104 samples (88.1%). About ninety-eight samples were of oncogenic high-risk HPV (HPV-16, HPV-18, HPV-31, HPV-33, HPV-35, HPV-45, HPV-52, HPV-58, and HPV-59), four samples were low-risk HPV (HPV-09 and HPV-53), and co-infection was observed in two samples. HPV-16, with sixty-three samples (53.3%), was the most prevalent, followed by HPV-18 with twelve samples (10.2%), HPV-35 with six samples (5.1%), HPV-45 with five samples (4.2%), HPV-52 with four samples (3.4%), HPV-9 with three samples (2.5%), HPV-33 with three samples (2.5%), HPV-31 with two samples (1.7%), HPV-58 with two samples (1.7%), and HPV-53 and HPV-59 with one sample (0.8%) each.

Squamous cell carcinoma was observed in 53 samples (84.1%) of HPV-16 and in 8 samples (66.7%) of HPV 18. Proportionally, adenocarcinoma was more frequent in HPV-18, where it occurred in three samples (25%), compared to HPV-16, where it was recorded in four samples (6.3%).

From a total of 63 samples for HPV-16, in 6 samples, it was not possible to identify the intratype variant, leaving 57 samples in which the variant was identified—33 samples (57.8%) of the A lineage, 2 samples (3.5%) from lineage B, 2 samples (3.5%) from lineage C, and 20 samples (35.0%) from lineage D.

Regarding age, the average was 48.3 years for the A lineage, 58.5 for the B lineage, 58.5 for the C lineage, and 46.4 for the D lineage. Squamous cell carcinoma was detected in forty-eight samples (84.2%), followed by adenocarcinoma in four samples (7.0%), of which three belonged to the D lineage. Lesions larger than 4.0 cm were the majority from 37 samples (64.9%). Clinical stage III was the most frequently observed stage out of 28 cases (49.1%) (Table 3). 

### 3.5. Cervical Cancer Treatment

Of the 118 patients, about 24 women (20.3%) underwent treatment with chemotherapy, teletherapy, and brachytherapy and were evaluated regarding their responses at their first follow-up visits. The treatment performed consisted of chemotherapy, based on cisplatin, at a dose of 40 mg/m^2^ for 6 weekly cycles, which is associated with conventional external radiotherapy (teletherapy). The dose varied between 45 Gy and 50.4 Gy in 25 to 28 h fractions with high dose-rate brachytherapy and a dose of 28 to 30 Gy at Point A. The treatment was generally well tolerated; the highest toxicity reported was gastrointestinal grade II in 12 patients (50.0%). There were few grade III reactions, which resulted in the necessity of halting treatment.

As for evaluating the type of HPV, twelve women had HPV-16, two women had HPV-18, four were HPV negative, and the rest, with one sample each, had HPV-9, HPV-31, HPV-33, HPV-35, HPV-58, and co-infection.

The return for the assessment of responses to treatment occurred at 110.3 days, on average. Due to the reduced number of patients, the categories for partial response, stable disease, and disease progression were unified to be considered a “present tumor”, while the complete response category was considered an “absent tumor”.

In this group of 24 patients, it was observed that 17 patients (70.8%) had a complete response to treatment on their first return visit. Ages ranged between 25 and 79 years, with a mean age of 52.5 years. A total of seven women (29.28%) had anemia, with a hemoglobin value below 10.0 g/dL, requiring a blood transfusion. About 20 specimens (83.3%) displayed squamous cell carcinoma. Tumors larger than 4.0 cm were the majority, at 13 cases (54.1%), and had a worse response to treatment than the group of patients with tumors measuring up to 4.0 cm. The analysis of the histological grade showed that the higher the grade, the greater the presence of a residual or recurrent tumor (Table 4). Of the 12 patients with HPV-16, a complete response was observed in 10 patients (83.3%). 

### 3.6. Treatment and HPV Lineages

**Figure 1:**
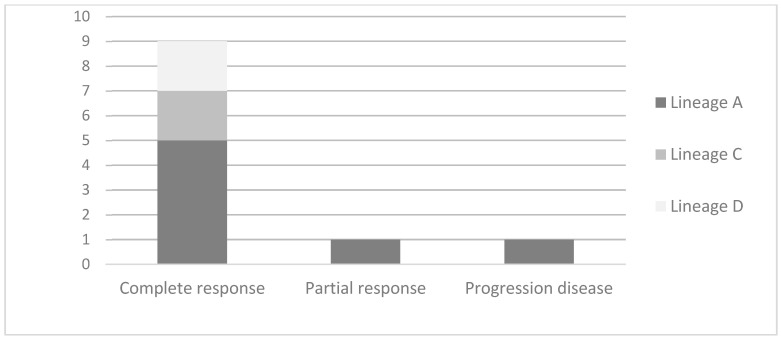
Local response of treatment after chemoradiotherapy in patients with human papillomavirus (HPV)-16 lineages.

Of the 57 women who were identified as having the HPV-16 variant, a total of 11 patients underwent chemotherapy, external radiotherapy, and brachytherapy treatment—seven were from the European group, carriers of the A lineage, and four from the non-European group, two from the C lineage and two from the D lineage. The age range varied between 25 and 76 years, with an average age of 46.5 years. Squamous cell carcinoma was the most frequent histological type and was observed in 10 women (90.9%). The tumors were well advanced in nine women (81.8%) and measured more than 4.0 cm. Clinical stage IIIB was the most frequent in seven patients (63.6%). An assessment of the response to treatment took place three months after its completion. A complete response occurred in nine patients (81.8%), five of whom were from the A lineage, two from the C lineage, and two from the D lineage (Figure 1). Cases of partial response and disease progression occurred in patients with the A lineage (Table 5).

## 4. Discussion

Among the works carried out in Brazil, the research carried out in Belém, Pará, stands out, where, in a population of 63 cases of invasive cervical cancer infected by HPV-16, 57 samples were obtained with the following lineage prevalence: A = 45.6%; B = 1.7%; C = 1.7%; and D = 50.8%. Also noteworthy is a cohort study carried out in Rio de Janeiro (RJ), where, in 334 samples of cervical cancer with the presence of HPV-16, the following frequency of strains was observed: A = 64.9%; B = 2.9%; C = 2.9%; and D = 29.0% [24,25]. In this study, which was carried out in São Luís, Maranhão, a total of 57 samples of HPV-16 whose lineages were identified were registered: 33 samples (57.8%) of the A lineage, 2 samples (3.5%) of the B lineage, 2 samples (3.5%) of the C lineage, and 20 samples (35.0%) of the D lineage. The percentage of variants for A and D was at intermediate levels in relation to the studies carried out in Pará and Rio de Janeiro, which could be the result of the miscegenation of the population in these states, as it is known that the Brazilian southeast region has a higher percentage of individuals of European origin, where a higher rate of variant A was observed, and the north of Brazil had a higher population percentage of those from a non-European origin. The indigenous population had the highest percentage of variant D. It is noteworthy that of all the studies carried out at a national or global level, only in Africa did variants B and C represent a significant percentage, meaning that a greater presence of these variants would be expected in populations with significant African ancestry, as in the case of Brazil.

The variability in the genetic material of HPV-16 variants could provide a difference in the prevalence of histological tumor types, which is similar to what occurs in the different types of HPV, where the frequency of adenocarcinoma is higher in HPV-18 than in HPV-16 [26]. A study that evaluated the prevalence of HPV-16 variants in cervical tumor samples from regions of Europe, Latin America, and Asia showed that, in squamous cell carcinomas, the percentages of lineage A varied between 83.7% and 97% and the percentages of lineages A and D varied between 3.0% and 16.3%; however, when we evaluated the adenocarcinomas, the A cell line varied from 36.6% to 67.9% and the D cell line varied between 28.6 and 63.3% [27]. In our samples, in the A lineage, the percentage of squamous cell carcinoma was 90.1%, and that of adenocarcinoma was 3.0%, whereas when we evaluated the D lineage, a prevalence of 75.0% in squamous cell carcinoma was observed, while this prevalence was 25.0% in adenocarcinoma. A limitation in our study was the non-classification into underlines, as we were uncertain as to which underline several samples would belong, reducing the casuistry, in addition to the fact that an increase in categories would make statistical analysis difficult. The largest study ever carried out with HPV-16 variants, which evaluated 3215 women with precursor lesions and invasive malignant lesions of the uterine cervix, demonstrated that differences could occur even within strains. When compared with the A1/A2 sublines, the A4 subline was associated with a 3.16-fold increased risk of developing cancer and a 9.8-fold increased risk of adenocarcinoma. By contrast, the D2 and D3 sublines, respectively, had a 137.3- and 59.4-times greater risk of developing adenocarcinoma than the A1/A2 sublines. Apparently, the A4, D2, and D3 sublines have a greater ability to infect glandular epithelial cells [28].

There is strong evidence showing that non-European HPV-16 variants have a higher oncogenicity due to their association with high-grade lesions of the cervix and invasive tumors [14,28,29,30,31,32]. Furthermore, it has been determined that some HPV-16 intratype variants can be preferentially associated with specific histological types of cancer [28,33]. Sublineage D was found to be more frequent in adenocarcinoma than in squamous cell carcinoma, for example [33].

Cisplatin-based chemoradiotherapy has become a cornerstone in the treatment of advanced cervical carcinoma over the last two decades. The quasi-simultaneous publication of randomized trials demonstrates an improvement in disease-free survival and overall survival [34,35,36]. Meta-analyses also observed better survival when compared to isolated radiotherapy [37]. After a complete course of radiotherapy, the lower the stage, the more likely it was that a complete response would be achieved—Stage I = 94%; Stage II = 86%; Stage III = 62% [38]. Complete response after a finished course of radiotherapy is a predictor of survival, and the 5-year survival rate varies between 76% and 41%, respectively, corresponding to whether a complete response is achieved or not [39].

Since patients with the same pathological factors do not react the same way to the same therapy and the human papillomavirus is the most common origin of all cervical cancers, it has been hypothesized that these types and variants could be used as prognostic factors to estimate an individual’s response to treatment and survival. The use of the HPV type as a prognostic factor remains controversial; some studies demonstrated a worse prognostic for HPV-18-infected patients [40], while other studies have demonstrated a poorer outcome for HPV-16 tumors [22,41]. HPV-16 is the most frequent type of human papillomavirus worldwide, according to normal cytology tests, which ranges from lesions at different stages of progression to cervical cancer, and it represents at least 50% of all cervical cancers in the world [14,42,43]. The study of variants and lineages of human papillomavirus is concentrated in HPV-16 variants and could be the key to understanding how differences in viral genomes influence the development of cancer and mediate its resistance to oncological treatment.

A few studies have addressed the association between HPV variants and their response to chemoradiotherapy. The literature suggests that European human papillomavirus variants have a better response to chemoradiotherapy. As Moreno-Acosta (2017) [44] reported in his previous work, eleven out of seventeen patients with the European variant, treated with radiotherapy +/− chemotherapy, achieved a complete response, and in the non-European variants, this complete response was observed in one of two cases. In our study, two of the seven patients with HPV-16 lineage A displayed tumor persistence; in the non-European HPV lineages, two patients with lineage C and two patients with lineage D were observed to have a complete response within three months of chemo-irradiation. 

Despite the literature registering a worse evolution in non-European strains of HPV-16, in our study, it was the European group that registered the worst response, with one case of partial response and another case of disease progression. The limited number of patients did not allow the degree of influence of the intratype variant to be precisely defined in response to treatment with chemotherapy and radiotherapy. Conducting studies with a larger number of patients could better answer this question. A major challenge that further studies still encounter is the situation of the state itself, as one of the poorest in the country, with a low HDI and few specialized services for cancer treatment that mostly serve a poor population. This causes delays and difficulty in accessing health care services with consequent worse results regarding the treatment of cancer.

## 5. Conclusions

Regarding the variants of HPV-16, A lineage dominated by 33 samples and squamous cell carcinoma was the most frequent of the four lineages. 

Among patients with HPV-16 variants who underwent treatment with chemotherapy, radiotherapy, and brachytherapy, it was observed that most achieved a complete response, with cases of partial response and disease progression occurring only in patients carrying the A lineage.

The small number of patients studied and the irregular distribution of lineages evaluated do not permit a stable association between HPV-16 lineages and their response to treatment. A multi-institutional cohort study, with a larger number of patients addressing various HPV-16 intratype variants, should resolve this issue.

## Figures and Tables

**Table 1:** Reproductive history and knowledge in terms of cervical cancer prevention in patients with cervical cancer, São Luís, Maranhão, 2016–2018, (*n* = 118).

Age of Onset of Sexual Activity (Years)	N	%
10–15	38	32.2%
16–18	37	31.5%
≥19	20	16.9%
Do not know/did not answer	23	19.5%
**Sexual partners**		
1	37	31.5%
2	26	22.0%
3	18	15.3%
4 or more	17	14.4%
Do not know/did not answer	20	16.9%
**Delivery number**		
0	04	3.4%
1 to 2	21	17.8%
3 to 4	29	24.6%
5 to 6	16	13.6%
7 to 8	22	18.6%
9 or more	24	20.3%
Do not know/did not answer	02	1.7%
**Knowledge about the purpose of the Pap test**		
Yes	87	73.7%
No	29	24.6%
Do not know/did not answer	02	1.7%
**Pap test before cancer diagnosis**		
Yes	102	86.4%
No	15	12.7%
Do not know/did not answer	01	0.8%
**Pap test frequency**		
Once a year	43	36.4%
Every 2 years	09	7.6%
Every 3 years	02	1.7%
Not regular	48	40.7%
Never	15	12.7%
Do not know/did not answer	01	0.8%

**Table 2:** Tumor staging and clinical aspects in patients with cervical cancer, São Luís, Maranhão, 2016–2018 (*n* = 118).

Tumor Staging		
EC 0	02	1.7%
EC IA1	01	0.8%
EC IB1	18	15.3%
EC IB2	01	0.8%
EC IIA	10	8.5%
EC IIB	30	25.4%
EC IIIB	50	42.4%
EC IVA	04	3.4%
EC IVB	02	1.7%
**Histological type**		
Squamous cell carcinoma	94	79.7%
Adenocarcinoma	11	9.3%
Carcinoma	08	6.8%
Malignant neoplasm	05	4.2%
**Tumor grade**		
Grade I	12	10.2%
Grade II	41	34.7%
Grade III	36	30.5%
Grade IV	07	5.9%
Not available	22	18.6%
**Tumor diameter**		
≥4.0 cm	49	41.5%
>4.0 cm	67	56.8%
Not available	2	1.7%
Lymph node metastasis		
Yes	15	12.7%
Not	60	50.8%
Not available	43	36.4%

**Table 3:** Clinical and pathological characteristics of patients with human papilloma virus (HPV)-16 lineages identified (*n* = 57).

HPV-16 lineages	A	B	C	D	*n* = 57	(%)
N° of patients	33	2	2	20	57	100.0
Mean age (years)	48.3	58.5	58.5	46.4		
**FIGO stage**						
IB1	5	0	0	3	8	13.9
IIA	4	0	0	2	6	10.5
IIB	7	0	1	3	11	19.3
IIIB	16	1	1	10	28	49.1
IVA	1	1	0	2	4	7.0
**Tumor size**						
≤4.0 cm	10	0	1	9	20	35.1
>4.0 cm	23	2	1	11	37	64.9
**Histological type**						
Squamous cell carcinoma	30	2	1	15	48	84.2
Adenocarcinoma	1	0	0	3	4	7.0
Carcinoma	1	0	0	1	2	3.5
Malignant neoplasm	1	0	1	1	3	5.2
**Grade of differentiation**						
Good	3	0	0	1	4	7.0
Moderate	10	1	0	8	19	33.3
Poor	12	1	2	6	21	36.8
Not reported	8	0	0	5	13	22.8

FIGO: International Federation of Gynecology and Obstetrics; HPV: human papilloma virus.

**Table 4:** Evaluation of response to treatment with chemotherapy + teletherapy + brachytherapy in patients with cervical cancer, São Luís, Maranhão, 2016–2018 (*n* = 24).

	Absent Tumor		Present Tumor		Total	*p*-Value
	*n*	(%)	*n*	(%)	*n* = 24	
**Age**						
20–29 years	1	50.0%	1	50.0%	2	0.8571
30–39 years	1	100.0%	0	0.0%	1	
40–49 years	6	85.7%	1	14.3%	7	
50–59 years	3	60.0%	2	40.0%	5	
60–69 years	4	66.7%	2	33.3%	6	
70–79 years	2	66.7%	1	33.3%	3	
**Hemoglobin levels**						
<10.0 g/dL	5	71.4%	2	28.6%	7	0.8576
>=10.0 g/dL	12	70.6%	5	29.4%	17	
**Histological type**						
Squamous cell carcinoma	15	75.0%	5	25.0%	20	0.604
Adenocarcinoma	1	50.0%	1	50.0%	2	
Malignant neoplasm	1	50.0%	1	50.0%	2	
**Tumor size**						
<=4.0 cm	9	81.8%	2	18.2%	11	0.2761
>4.0 cm	8	61.5%	5	38.5%	13	
**Histological grade**						
Grade I	2	100.0%	0	0.0%	2	0.3788
Grade II	5	62.5%	3	37.5%	8	
Grade III	4	57.1%	3	42.9%	7	
Grade IV	1	50.0%	1	50.0%	2	
Not available	5	100.0%	0	0.0%	5	

**Table 5:** Evaluation of response to treatment with chemotherapy + teletherapy + brachytherapy in patients with cervical cancer and carriers of HPV-16 variants, São Luís, Maranhão, 2016–2018 (*n* = 11).

	HPV-16 Lineages				
				*n* = 11	(%)
**Age**	**16 A**	**16 C**	**16 D**		
Medium age (years)	44.4	58.5	42.0		
**Histological type**	**16 A**	**16 C**	**16 D**		
Squamous cell carcinoma	7	1	2	10	90.9%
Malignant neoplasm	0	1	0	1	9.1%
**Histologic grade**	**16 A**	**16 C**	**16 D**		
Grade II	2	0	2	4	36.4%
Grade III	1	1	0	2	18.2%
Grade IV	0	1	0	1	9.1%
Not available	4	0	0	4	36.4%
**Tumor diameter**	**16 A**	**16 C**	**16 D**		
Until 4.0 cm	1	1	0	2	18.2%
>4.0 cm	6	1	2	9	81.8%
**Stage**	**16 A**	**16 C**	**16 D**		
EC IIA	2	0	0	2	18.2%
EC IIB	1	1	0	2	18.2%
EC IIIB	4	1	2	7	63.6%
**Response evaluation**	**16 A**	**16 C**	**16 D**		
Complete	5	2	2	9	81.8%
Partial	1	0	0	1	9.1%
Disease progression	1	0	0	1	9.1%

## Data Availability

All data are included in this manuscript.
